# Potential capacity of interferon-α to eliminate covalently closed circular DNA (cccDNA) in hepatocytes infected with hepatitis B virus

**DOI:** 10.1186/s13099-021-00421-9

**Published:** 2021-04-12

**Authors:** Gang Wang, Jun Guan, Nazif U. Khan, Guojun Li, Junwei Shao, Qihui Zhou, Lichen Xu, Chunhong Huang, Jingwen Deng, Haihong Zhu, Zhi Chen

**Affiliations:** 1 State Key Laboratory for Diagnosis and Treatment of Infectious Diseases, Collaborative Innovation Center for Diagnosis and Treatment of Infectious Diseases, The First Affiliated Hospital, College of Medicine, National Clinical Research Center for Infectious Diseases, Zhejiang University School of Medicine, 79 Qingchun Road, Hangzhou, 310003 Zhejiang China; 2 Institute for Hepatology, Shenzhen Third People’s Hospital, National Clinical Research Center for Infectious Disease, Shenzhen, 518112 Guangdong China; 3 The Second Affiliated Hospital, School of Medicine, Southern University of Science and Technology, 518112 Shenzhen, China

**Keywords:** Hepatitis B virus, interferon-α, Covalently closed circular DNA (cccDNA), Chronic HBV infection, Deamination

## Abstract

Interferon-alpha (IFN-α) and nucleot(s)ide analogs (NAs) are first-line drugs for the treatment of chronic hepatitis B virus (HBV) infections. Generally, NAs target the reverse transcription of HBV pregenomic RNA, but they cannot eliminate covalently-closed-circular DNA (cccDNA). Although effective treatment with NAs can dramatically decrease HBV proteins and DNA loads, and even promote serological conversion, cccDNA persists in the nucleus of hepatocytes due to the lack of effective anti-cccDNA drugs. Of the medications currently available, only IFN-α can potentially target cccDNA. However, the clinical effects of eradicating cccDNA using IFN-α in the hepatocytes of patients with HBV are not proficient as well as expected and are not well understood. Herein, we review the anti-HBV mechanisms of IFN-α involving cccDNA modification as the most promising approaches to cure HBV infection. We expect to find indications of promising areas of research that require further study to eliminate cccDNA of HBV in patients.

## Background

For approximately 60 years, it has been known that hepatitis B is caused by a viral infection, and the hepatitis B virus (HBV) has been definitively identified as the causative factor [1]. Over time, an increasing details have been discovered in regards to hepatitis B, and is now well known that HBV is a hepadnavirus. More specifically, HBV is a hepatotropic DNA virus approximately 42 nm in diameter with particles consisting of surface proteins and core proteins [2] surrounding a partially double-stranded, relaxed, and circular 3.2 kb genomic DNA component (called rcDNA) [3] (additional details can be found in other reviews [1, 4]). During its natural life cycle, HBV enters hepatocytes by attaching to surface proteins, such as hepatocyte-associated heparan sulfate proteoglycans (HSPG) and sodium taurocholate cotransporting polypeptide (NTCP) [5]. Next, the core particle (nucleocapsid) of HBV is released into the cytoplasm [6]. The genomic rcDNA of HBV is released from the nucleocapsid and, through the nuclear pore complex, enters into the nucleus of the hepatocyte [7]. Once inside the nucleus, the gap in the rcDNA is repaired by the DNA repair machinery of the host cell to generate the covalently closed circular DNA (cccDNA) [6–8]. Importantly, this cccDNA is responsible for difficulty in curing the HBV infections, which can lead to chronic liver inflammation, cirrhosis, and even hepatocellular carcinoma [6, 9, 10].

Once formed in the cell nucleus, cccDNA is very difficult to get eliminated from infected cells and can persist continuously until the death of the host cell [11]. cccDNA can form a dynamic pool within the nuclei, where it supports its persistence and encodes its structural proteins (including surface proteins, core proteins), polymerases and the X protein [6]. According to the guidelines from the European Association for the Study of the Liver, the American Association for the Study of Liver Diseases, and the Chinese Society of Hepatology and the Chinese Society of Infectious Diseases, interferon (IFN-α) and nucleotide analogues (NAs) are recommended, as needed, for the treatment of HBV infection in patients [12–14]. In addition to IFN (pegylated interferon-alpha, [PegIFNα]), the following are also used for the inhibition of viral replication: entecavir (ETV), tenofovir disoproxil fumarate (TDF), adefovir (ADF), lamivudine (LAM) and telbivudine (LDT) are used for the inhibition of viral replication [12–14]. Although NAs have been shown to be efficient for decreasing the viral protein and DNA loads in the serum of HBV-infected patients and effectively hinder the progression of chronic HBV infection, cccDNA is the most difficult obstacle preventing a complete cure of HBV since it is not effectively and directly eliminated by current drugs [15–18]. Until now, IFN-α was the only potential candidate against persistent cccDNA in the nuclei of the hepatocytes in patients with chronic HBV infections [11, 19–21].

Based on basic research indications, IFN-α might be one of the most promising treatments for the elimination of HBV cccDNA. However, clinical evidence has shown that IFN-α treatment has limited efficacy against chronic infections in HBV-positive patients [22]. Unfortunately, there is insufficient information to clarify why the IFN-α mechanisms of action are ineffective for the treatment of HBV. Herein, the details of IFN-α on cccDNA that persists in the hepatocytes of HBV-infected patients are discussed in hopes of uncovering even a small insight that would lead to an effective treatment or even a cure for chronic HBV infections.

## Importance of cccDNA in HBV life cycle

**Fig. 1:**
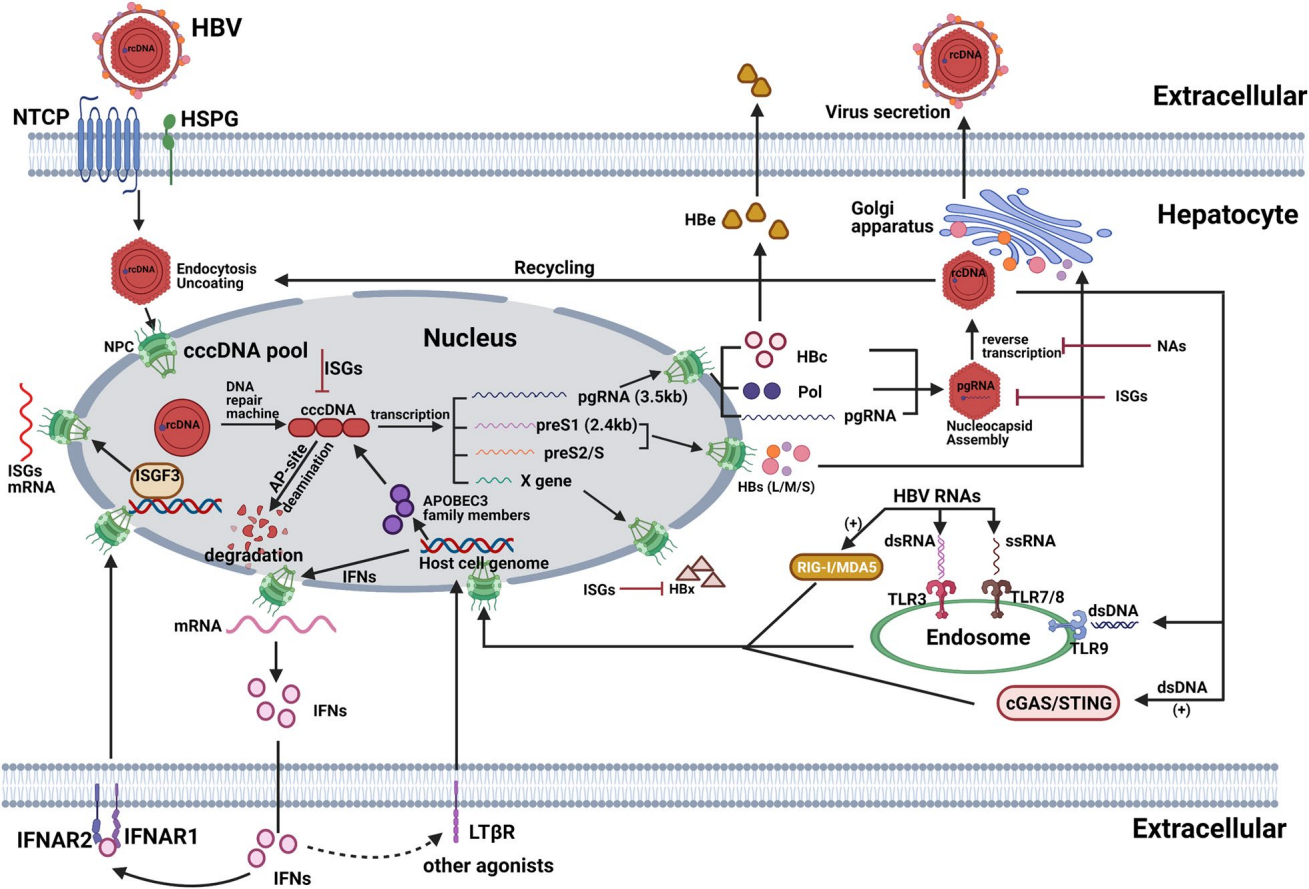
Life cycle of HBV and antiviral mechanisms of cellular innate immunity in hepatocyte. During the lifecycle of HBV, nucleic acid (RNA and DNA) can trigger innate immune system sensors (cGAS/STING, TLRs, or RIG-I) which then initiate a cascade of antiviral signaling. As a result, STATs and IRFs enter into nucleus to promote the transcription of effector genes. The most important effector proteins, IFNs, are secreted out of the cell and bind to receptors (IFNRs) on the cell surface, which then drive hundreds of ISGs to target multiple steps in the lifecycle of HBV. APOBEC3 family members activated by lym. *NPC* Nuclear pore complex, *NTCP* sodium taurocholate cotransporting polypeptide, *HSPG* hepatocyte-associated heparan sulfate proteoglycans, *cccDNA* covalently closed circular DNA, *pgRNA* pregenomic RNA, *Pol* HBV polymerase, *HBs(L/M/S)* HBV surface antigen (large, middle, small), *HBc* HBV core antigen, *HBx* HBV x protein, *HBe* HBV e antigen, *NAs* nucleotide analogs, *IFN* interferon, *ISGs* IFN stimulated genes, *TLRs* toll like receptors, *RIG-I* retinoic acid inducible gene I, *MAD5* Melanoma differentiation-associated protein 5, *cGAS* cyclic GMP–AMP synthase, *STING* Stimulator of interferon genes. (+) stands for “activation”. (Figure created with BioRender (https://biorender.com))

It is necessary to understand the details of the HBV life cycle to fully comprehend the mechanisms of an anti-HBV treatment (Fig. 1). As a member of Hepadnaviridae family of viruses, HBV has a 42 nm diameter (Dane particle) and spherical structure, and it consists of viral surface antigens (HBsAgs), including large, middle and small surface glycoproteins, and a lipid envelope. These components help the virus enter into hepatocytes via binding to receptors and fusing with the cell membrane [23]. Within the Dane particle (virion), under the outer proteolipid envelope, there is an icosahedral nucleocapsid core composed of HBV core antigen (HBcAgs) that surrounds both the DNA polymerases as well as the circular, partially double stranded DNA [24]. Upon infection, binding of the small surface antigen (SHBs) protein to the HSPG and the binding of the large surface antigen (LHBs) to NTCP are essential and sufficient for HBV attachment to a hepatocyte [5, 25]. Through protein-mediated endocytosis, HBV particles enter hepatocytes, and the fusion of the HBV envelope with the membrane of an endosome triggers the release of the capsid into the cytoplasm [26]. Following an interaction with microtubules, the viral capsid is then transported into the nucleus through the nuclear pore complex (NPC) [27]. The polymerase or the capsid protein contains a nuclear localization signal (NLS) that enables the rcDNA to localize to the nucleus with the help of importin α and importin β [28]. Once in the nucleoplasm, the rcDNA is repaired by the associated factors of the host cells in the final process of completing the cccDNA. After the short RNA primer and polymerase protein are removed, the minichromosome persists long-term in the nucleus [29]. cccDNA is a template in the nucleus that can be transcribed by the host RNA polymerase II into four types of HBV transcripts: pregenomic RNA (pgRNA, 3.5 kb), pre-S1 RNA (2.4 kb), pre-S2/S RNA (2.1 kb) and the X gene (0.7 kb). These transcripts are then translated into HBV core proteins and a viral polymerase, large surface proteins, middle and small surface proteins and the HBx protein in the cytoplasm, respectively [30]. The formation of a complex consisting of pgRNA and a polymerase initiates nucleocapsid assembly using core HBV protein dimers as building blocks, and during this process, the P protein itself primes the reverse transcription of the pgRNA to synthesize the minus strand of HBV rcDNA. Subsequently, the plus-strand of the rcDNA is synthesized by the P protein in a DNA-dependent manner [31]. The current anti-HBV drugs, including NAs (lamivudine, adefovir, telbivudine, entecavir, and tenofovir), all target this process that primes, initiates, or elongates new synthesis [31]. With the RNase H activity of the P protein, pgRNA can be degraded during the formation of minus-strand [32]. The nucleocapsid containing the rcDNA acquires a lipid bilayer with HBV surface antigens to form the viral envelope, and the viruses are trafficked and secreted outside the hepatocytes, possibly with the help of the endosomal sorting complex required for transport (ESCRT) protein [33]. Although this is a brief depiction of the HBV life cycle, most of the current drugs effectively target the reverse transcription step; however, new drugs with novel targets are desired for clinical application.

## Characteristics of nuclear cccDNA

During the HBV life cycle, viral rcDNA is translocated to the nucleus of the host cell, where the cccDNA persists after a complicated process in which the viral polymerase is removed from the 5′-end of the negative DNA strand, a short RNA oligomer is used as a primer for DNA synthesis of the sense strand, the redundant sequence of the viral DNA is removed, and the cccDNA is formed after a ligation performed by the cellular replication machinery of the host [11, 34]. cccDNA in the nucleus is a constant transcriptional template for pgRNA, which is subsequently reverse transcribed into viral genomic DNA packages in the nucleocapsid for HBV assembly [35]. In addition, the mRNA of the surface, core and e-antigens, as well as the X protein are all transcribed from the cccDNA in the nucleus [6].

The initial nuclear cccDNA originates from the viral infection; however, the major sources of cccDNA in the nuclear pool are the newly reverse-transcribed rcDNA in the nucleocapsids [36]. Further, the size of the cccDNA pool, which is formed at the early stage of infection, ranges from 30 to 50 copies of cccDNA per cell [37, 38]. Within the pool, the half-life of the cccDNA varies between different cell types; the half-life is longer than 30 days in stable hepatocytes and fewer than 5 days in unstable hepatocytes [39, 40].

The stable structure of cccDNA in the nucleus is the minichromosome, also called the episome, which is extrachromosomal DNA [41, 42]. Using the duck liver virus model and HepG2.2.15 (HepG2 cells with stable HBV expression), two separate research groups from the USA and Germany discovered histone proteins that coprecipitated with the viral genome and a 180 bp fragment of the HBV genome was detected when the samples were enzymatically digested [41, 42]. In eukaryotic cells, the predominant length of the chromatin fragments was 200 bp, and compared with the DNA of host cells, the digested HBV genomic fraction was subsequently shorter [43]. With respect to the arrangement, a HBV genomic minichromosome was visualized under an electron microscope and was found to resemble beads-on-a-string [42]. Similar to those of the host cells, histone proteins are essential components of the minichromosome and can bind tightly to cccDNA. With the help of HBV core proteins, the minichromosome is supercoiled with histones, where histone H3 and H2b are predominant, while H2A, H4 and H1 are less commone [11, 44]. Similar to standard histone function, these histones are involved in the regulation of viral cccDNA gene expression [45]. Histones H3 and H4 can be acetylated or deacetylated, resulting in the suppression or enhanced expression of HBV genes [45].

## The potential capacity of IFN-α to eliminate cccDNA

### The IFN family: a brief overview

IFNs are a family of cytokines, including INF-α, IFN-β, INF-γ, INF-ω and others, as well as newly identified members (IFN-λ [46]). The family members can be generally classified into three types: type I, type II and type III (see detailed reviews [47–49]). Type I IFNs, which include IFN-α, IFN-β and others, can be synthesized and secreted by most cell types that are infected by viruses. There is only one type II IFN, IFN-γ, and it is usually only produced only by immune cells, including natural killer (NKs) cells, CD4^+^ T helpher cells and CD8^+^ cytotoxic T lymphocytes (CTLs) [47]. In 2003, a new interferon (IFN- λ [a type III IFN]) was independently reported by two groups [50, 51]. Type III IFNs, including IFN-λ (IL-28 A), IFN- λ2 (IL-28B) and IFN-λ3 (IL-29), are similar to type I IFNs, in that they can be produced by most cells infected by viruses [50, 51].

All type I IFNs bind to a common receptor, which is a heterodimer consisting of two proteins: interferon-α receptor 1 (IFNAR1) and 2 (IFNAR2) [47]. IFN-γ (type II) also binds to a heterodimeric receptor comprised of two subunits: interferon gamma receptor 1 (IFNGR-1) and IFNGR2 [47]. In constrast, although the receptor for type III IFNs is also a heterodimer, only one subunit is specific to type III IFNs: interferon lambda receptor 1 (IFNLR1) forms a complex with a shared receptor subunit, interleukin-10 receptor (2IL10R2) [50–52]. Once an IFN receptor is bound and the signaling pathway is activated, all IFNs induce the recruitment and phosphorylation of Janus-activated kinases/signal transducers and activators of transcription (JAK/STAT) signaling pathway components, which ultimately leads to the nuclear translocation of various proteins to induce the expression of downstream genes (see detailed reviews [47, 48, 52]).

Importantly, only IFN-α has been commonly used for the treatment of chronic HBV infection in clinical practice, including IFN-α-2a (Pegasys, Roche) and IFN-α-2b (PegIntron, MSD) [12–14, 53].

As mentioned above, IFN-α binds to IFNAR-1/2 heterodimer to activate the JAK/STAT signaling pathway [47]. IFNAR-1 and IFNAR-2 subsequently phosphorylate tyrosine kinase2 (Tyk2) and JAK1, respectively [47]. Subsequently, STAT1 and STAT2 can also be activated through phosphorylation [47]. The phosphorylation of STAT1 and STAT2 leads to the formation of STAT1/2 heterodimers, which can translocate from the cytosol into the nucleus. Once in the nucleus, STAT1/2 can bind to interferon regulatory factor (IRF) 9, a member of the IRF family, to form a complex with IFN-stimulatory gene factor 3 (ISGF-3). This complex, binds to IFN-stimulatory response element (ISRE) sequence close to the promoter region of the IFN-regulatory genes (see detailed reviews [47, 54, 55]). In this regulatory complex, IRF9 has the ability to bind to the ISRE of target genes and STAT1/2 can recruit other factors to initiate the transcription of genes that defend against viral infections [56]. Additionally, IFN-α can signal through mitogen-activated protein kinase (MAPK) signaling pathways to promote the transcription of numerous other associated genes [57]. Of the MAPKs pathways, p38-mediated signaling involves IFN-α triggering IFN-stimulated genes in a STATs-independent manner [57]. Two proteins, Rac (a Rho GTPase) and Vav (the Rac1 guanine nucleotide exchange factor), bridge the activation of the IFN and p38 MAPK signaling pathway cascades [57].

### Antiviral mechanism of IFN-α

The standard means to induce the expression of IFN-α have already reviewed in detail [20]. Once a virus enters a host cell, viral nucleic acid sensor proteins, such as cyclic GMP–AMP synthase/stimulator of interferon genes (cGAS/STING), Toll-like receptor (TLRs)-3, -7, -8 and -9 or retinoic acid inducible gene I (RIG-I)/Melanoma differentiation-associated protein 5 (MAD5), recognize viral DNA or RNA. Upon this recognition, antiviral signaling cascades are initiated, and IFN is produced, one of the most important response proteins [58]. Hundreds of IFN-stimulated genes (ISGs) have been identified in recent decades. Among the ISGs, some well-known proteins and classical ISGs have been shown to exert antiviral effects, including IFN-inducible dsRNA activated protein kinase (PKR), 2′-5′-oligoadenylate synthetase (OSA)/RNase L, adenosine deaminase acting on RNA (ADAR1), myxovirus resistance 1 (MxA/Mx1), inducible nitric oxide synthase (iNOS) and major histocompatibility complexes (MHC) I and MHC II [47, 49, 59]. Subsequent to these discoveries, many ISGs were identified using high throughput screening, including IRF1, RIG-I, MDA5, DExD/H-box helicase 60 (DDX60), IFN-induced transmembrane proteins (IFITMs), APOBEC3 family proteins, tripartite motif-containing protein 5 alpha (TRIM5α), and others [60, 61].

ADAR1 is an IFN-inducible dsRNA-specific adenosine deaminase that can recognize RNA or DNA [47]. Activated ADAR1 can hydrolyze the amino group at the C-6 position of adenosine to yield inosine (adenine to hypoxanthine) [62, 63]. This conversion destabilizes the 3D structure of the dsRNA, and the hypoxanthine is recognized by guanine during transcription [47]. Furthermore, ADAR1 can recognize Z-DNA, a type of dsDNA with a double helical structure; however, the functional significance of Z-DNA has not been determined [64]. PKR, found in both the cytosol and nucleus, can be activated by dsRNA to self-phosphorylate. In association with ribosomes, activated PKR has the capacity to phosphorylate several other proteins, some of which are involved in the translation of viral mRNA [47]. OAS also recognizes and is activated by dsRNA, where it then catalyzes ATP to 2′-5′-oligo adenylic acid, which consequentially activates RNase L (a latent cytosol endonuclease) to degrade the invading viral dsRNA [47]. MxA, also known as Mx1, is a GTPase that can block the replication of individual viruses through its GTPase activity [47, 65]. The induction of NOS as well as MHC I and MHC II expression has been implicated in the antiviral activity of immune cells [47]. APOBEC3 family members and other ISGs will be discussed in upcoming sections. Additionally, as IFN-α is the focus, it will therefore be emphasized in the remainer of the review.

## IFN-α mechanisms of action against HBV infection

### Clinical use of IFN-α for treatment of HBV infection

The use of IFN-α and NAs in combination has been recommended for the treatment of chronic HBV infections [12–14]. This treatment leads to a 3–7% loss of HBsAgs in patients, a clinical outcome that has not been satisfactory for patients [13]. The reason for these clinical failures might be the persistence of the cccDNA in the nuclei of hepatocytes [10].

Although the clinical use of NAs for the treatment of chronic HBV has made considerable progress, many patients treated with NAs experience only short-term or momentary relief from the serum load of HBV products [66]. The reason for this unsatisfactory result is the persistence of the HBV cccDNA. IFN-α, especially pegIFN-α, is still one of the most important alternatives for the treatment of chronic HBV, according to the worldwide clinical guidelines [12–14]. Among the treatment approaches currently used, IFN-α is the only potential candidate with the capacity to eliminate cccDNA in the nucleus of infected hepatocytes. The molecular mechanisms by which IFN-α defends against cccDNA are subsequently described, and a perspective is offered regarding how IFN-α can contribute to curing chronic HBV in the future. HBV replication has been shown to be dramatically decreased by IFN-α in vitro and in vivo [19, 67].

Nonetheless, it is important to note that the long-term administration of IFN-α does not have the capacity for complete seroconversion in chronic HBV infection patients without elimination of the cccDNA; only 38.8% of patients experienced HBeAg seroconversion, and 61.2% of patients in this cohort had no response [68].

### IFN-α regulates the transcription of cccDNA

In the HBV genome, there is also an IFN-α response element, the enhancer 1/promoter region of the HBx gene, which can be inhibited by IFN-α-induced factors under mild conditions [67, 69]. Additional reports have described that a sequence, from nucleotide 1091 to 1100, has the characteristics of an ISRE/IRE and can bind IGF3 and IRF1, thereby moderately regulating the transcription of HBV genes [69].

Since the cccDNA and histones in the nucleus form episomal minichromosomes, the epigenetic modifications of the histones influences the expression of the HBV genes [70]. In primary human hepatocytes (PHH) infected with HBV in vitro, treatment with IFN-α dramatically increased the expression of the IFIT1 gene, which is an ISG, and in turn led to low levels of K4 methylation, K27 acetylation, and K122 acetylation in histone H3, modifications that inhibited transcription of the cccDNA genes [70].

Using ChIP, histone H4 of the cccDNA minichromosome was found to be hypoacetylated in an HBx protein-independent manner after 48 h of treatment with IFN-α in cultured cells or chimeric albumin enhancer/promoter-driven urokinase-type plasminogen activator/severe combined immunodeficient (uPA/SCID) mice [19]. In addition to histone H4, histone H3 bound with cccDNA can also be acetylated to regulate the transcriptional activity of the cccDNA [45]. IFN-α treatment enhances the recruitment of deacetylases, including histone deacetylase 1 (HDAC1), sirtuin-1 (Sirt1), enhancer of zeste homolog 2 (Ezh2) and Ying Yang 1 (YY1), for the essential function of the cccDNA IRSE [19]. Additionally, the IRSE of the minichromosome induces the binding activity of STAT1 and STAT2, the phosphorylation of which was decreased [19].

Additionally, several recently published reports have confirmed the effects of IFN-α on epigenetic modifications of cccDNA leading to the repression of HBV. Transcriptional activity of the cccDNA is maintained by the succinylation of histone H3K79 of the minichromosome [71]. IFN-α was shown to suppress the general control non-repressed 5 (GCN5) protein, a histone acetyltransferase with succinyltransferase activity, which decreased of succinylation of H3K79 and lead to clearance of HBV cccDNA [71]. Several genes, such as STAT1, promyelocytic leukemia protein (PML), and structural maintenance of chromosomes flexible hinge domain containing 1 (SMCHD1), have epigenetic modification capabilities and can reduce acetylation levels of histione H3K9 and H3K27 of the minichromosome, resulting in decreased levels of cccDNA [72]. Furthermore, IFN-inducible protein 16 was reported to epigenetically modify H3K4, H3K9 and H3K27, which suppressed the cccDNA [73].

### IFN-α indirectly targets cccDNA through APOBEC3 family proteins

The antiviral activity of the activation-induced cytidine deaminase (AID)/apolipoprotein B mRNA editing enzyme, in the catalytic polypeptide-like (APOBEC) family of proteins, including APOBEC1, APOBEC2, APOBEC3, APOBEC4 and AID (reviewed elsewhere [74–76]), has been reported to initially suppress the human immunodeficiency virus through hypermutations that result in the deamination of enzymes [77–80]. Within the APOBEC family, APOBEC3 members have the potential for anti-HBV activity. Bonvin et al. showed that IFN-α induces the expression of APOBEC3B/3C/3F/3G in primary hepatocytes and cell lines (HepG2 and Huh7) and, of these four proteins, APOBEC3B (the long form), APOBEC3F and APOBEC3G can suppress HBV replication by deaminating HBV replicative intermediates, possibly including cccDNA, although it was not specifically identified [81]. At approximately the same time, another research group obtained the same results and confirmed that APOBEC3B/C/F/G were capable of mutating the minus-strand, and three of these proteins, APOBEC3B/F/G, were also capable of mutating the plus-strand of the HBV genome [82]. A previous report also confirmed that APOBEC3G is involved in the inhibition of HBV replication partially due to a suppression of HBV pgRNA packaging, and the accumulation of HBV DNA was prevented in the presence of APOBEC3G [83]. Approximately one decade later, in 2014, Lucifora et al. searched for the key proteins responsible for the elimination of cccDNA following IFN-α treatment and found that the activation of the lymphotoxin (LT)-β receptor, a TNF receptor superfamily member, leads to cccDNA degradation through the deamination of APOBEC3A or APOBEC3B, which gave rise to apurinic/apyrimidinic (AP) sites in the cccDNA [84]. Additionally, heat shock proteins (Hsp), in particular Hsp90, have the potential to increase the deamination activity of APOBEC3G, which ultimately results in HBV genome mutations in HepG2 cells [85]. In chronic HBV-infected patients, IFN-α treatment increases the expression of all the APOBEC3 proteins, but especially APOBEC3A and APOBEC3B [86].

In the reports above, although changes to the HBV genome by deaminases had been found in cell lines, all genomic DNA had been targeted, including HBV rcDNA and cccDNA as well as the cell genomic DNA [82]. Next, Nair et al. sought to confirm the applicability of these results, but the data did not support the deamination of the genomic DNA due to the capsid protein [87]. These results consistently suggest that the APOBEC3 proteins suppress HBV replication; however, whether the molecular events are directly related to cccDNA or not remains unknown. Direct evidence linking IFNs, APOBEC3 and cccDNA degradation should be explored in the future. Deaminases are highlighted as prospects for the elimination of cccDNA.

As a cytokine, IFN-α is capable of modulating innate and adaptive immune cells, which results in the indirect elimination of the cccDNA [88]. Other cytokines, such as transforming growth factor (TGF-β), IFN-γ and tumor necrosis factor (TNF)-α, can also facilitate the degradation of cccDNA in hepatocytes, in a manner similar to that of IFN-α, upon induction by proteins or enzyme, such as APOBEC3A/B and others [89, 90]. Thus, further investigation should focus on finding new proteins or targets of HBV infection.

Deamination enzyme and nuclease are powerful and bright prospective tools for the complete removal of cccDNA in the future. For instance, ISG20, an IFN-induced exonuclease that can degrade viral nucleic acids, including DNA and RNA, was shown to be involved in viral clearance in chimpanzees as well as the degradation of HBV RNA [91, 92]. Consequently, deamination and IFN-induced nuclease are two promising directions of investigation regarding a cure for HBV infections.

### Recent findings of poor clinical responses to IFN-α

IFN-α has been proven to be insufficient for the treatment of patients with chronic HBV infection [93]. The evidence suggests that components translated from the HBV genome interact with and block proteins of the IFN-α signaling pathway, ultimately counteracting the effects of IFN-α [94–96]. The key cellular process of the IFN-α-induced signaling pathway, the translocation of STAT family members into the cell nucleus, can induce and stimulate the expression of several downstream genes, which in turn exert antiviral effects. Chen et al. reported that the products of the polymerase open reading frame of the HBV genome inhibited the phosphorylation of protein kinase C (PKC)-δ, which further modified the phosphorylation status of a serine residue in STAT1 [94]. Additionally, HBV polymerase can block the binding of importin-α5, which promoted the translocation of the STAT1/2 dimer into the cell nucleus to induce the expression of antiviral genes [94]. Later, Chen et al. proposed that groups of HBV genome splice variants, specifically splicing-protein 1 (SP1), SP2 and SP3, disrupt the activation of the JAK/STAT pathway and attenuate the effects of IFN-α treatment [96]. Furthermore, HBV surface antigens inhibit the phosphorylation of a serine residue in STAT3, and decrease levels of APOBEC3G [84, 97]. The HBx protein has also been reported to transcriptionally induce the expression of suppression of cytokine signalling 3 (SOCS3) and protein phosphatase 2 A (PPA), which are negative regulators of the IFN-α-induced JAK/STAT signaling pathway [98]. The clinical anti-HBV effects of IFN-α have been detailed previously [22, 99, 100]; however, these poor clinical responses should be further explored to determine additional and more detailed mechanisms that may lead to more efficient clinical use of IFN-α.

Recently, an ISG called the Mx2 protein was described as restricting the cccDNA pool by disturbing the conversion of rcDNA to cccDNA [101]. Further, it has been reported that the rs59391722 allele exists in the promoter region of the ubiquitin conjugating enzyme E2 L3 (UBE2L3) gene resulting in the degradation of APOBEC3A, leading to an increased sensitivity to HBV infection in adults and children [102]. APOBEC3 family members should be further explored with a focus placed on modification of HBV cccDNA in hepatocytes.

## Conclusions

HBV infection remains a global health problem due to the persistence of a chronic infection that can lead to severe conditions, such as liver fibrosis, liver cirrhosis, and even hepatocellular carcinoma [12–14, 103].

Significant clinical progress has been made in the development of vaccines for the prevention of HBV infection and in regard to IFN-α and NAs for the treatment of chronic HBV infection [12–14]. In terms of the cellular and molecular aspects, the details of the HBV life cycle have been illustrated comprehensively: virus attachment via receptors, fusion with the cell membrane and endocytosis, and release; transportation of the nucleocapsid along cellular microtubules; binding to the nuclear transport complex and release of rcDNA into nucleus; the formation of cccDNA; the regulation of transcription, the pgRNA capsid and reverse-transcription; formation of the nucleocapsid; and release of new viruses and the recycling of rcDNA and cccDNA [104, 105]. Throughout the course of the HBV life cycle, many processes and components can be targets for drug development, such as the transcription of cccDNA and reverse transcriptase [106, 107]. Notwithstanding the continuous anti-HBV action of the available drugs to prevent or delay the events of end-stage liver diseases, the persistence of cccDNA in the liver cells of patients with chronic HBV remains the biggest challenge to developing a cure [12–14]. Currently, the clinically available drugs for chronic HBV infections include NAs and/or IFN-α [12–14]. It is known that, thus far, the cccDNA pool in hepatocytes has been very difficult to eliminate, and use of IFN-α has a potential to target cccDNA [19, 81, 84]. Although the expected results of using IFN-α to target cccDNA have been reported in experiments using cell lines and animal models, the clinical effectiveness of the cytokine has not been as efficacious as the in vitro and in vivo studies have suggested [22, 84]. The mechanisms of these outcomes are still not clear. In the future, research should be focused on the exploration of the paradoxical results of the laboratory and the clinic, which facilitate the development of cure for chronic HBV infections.

## Data Availability

Not applicable.

## Abbreviations

HBV: Hepatitis B virus
cccDNA: Covalently closed circular DNA
IFN-α: Interferon-α
NAs: Nucleot(s)ide analogs
rcDNA: Relaxed circular DNA
HSPG: Hepatocyte-associated heparan sulfate proteoglycans
NTCP: Sodium taurocholate cotransporting polypeptide
PegIFNα: Pegylated interferon-α
ETV: Entecavir
TDF: Tenofovir disoproxil fumarate
ADF: Adefovir
LAM: Lamivudine
LDT: Telbivudine
HBsAgs: Viral surface antigens
HBcAgs: HBV core antigens
SHBs: Small surface antigen
LHBs: Large surface antigen
NPC: Nuclear pore complex
NLS: Nuclear location signal
pgRNA: Pregenomic RNA
JAK/STAT: Janus-activated kinases/signal transducers and activators of transcription
IRF9: Interferon regulatory factor 9
ISGF-3: IFN-stimulatory gene factor 3
ISRE: IFN-stimulatory response element
cGAS/STING: Cyclic GMP–AMP synthase/stimulator of interferon genes
RIG-I/MAD5: Retinoic acid inducible gene I/Melanoma differentiation-associated protein 5
APOBEC: Activation-induced cytidine deaminase (AID)/apolipoprotein B mRNA editing enzyme, in the catalytic polypeptide-like
SOCS3: Suppression of cytokine signalling 3
UBE2L3: Ubiquitin conjugating enzyme E2 L3
